# Multimodal Gold Nanostars as SERS Tags for Optically-Driven Doxorubicin Release Study in Cancer Cells

**DOI:** 10.3390/ma14237272

**Published:** 2021-11-28

**Authors:** Luca Minati, Devid Maniglio, Filippo Benetti, Andrea Chiappini, Giorgio Speranza

**Affiliations:** 1 IMMAGINA BioTechnology, Via Sommarive 18, 38123 Trento, Italy; 2 Department of Industrial Engineering, University of Trento, Via Delle Regole 101, 38123 Trento, Italy; devid.maniglio@unitn.it (D.M.); fbenetti86@gmail.com (F.B.); speranza@fbk.eu (G.S.); 3 BIOtech Research Centre, University of Trento, Via Delle Regole 101, 38123 Trento, Italy; 4 Institute of Photonics and Nanotechnologies (IFN-CNR-IFN), Via Alla Cascata 56/C, Povo, 38123 Trento, Italy; andrea.chiappini@unitn.it; 5 Fondazione Bruno Kessler, Via Sommarive 18, 38123 Trento, Italy

**Keywords:** gold nanostars, SERS, drug release, in vitro cell imaging

## Abstract

Surface Enhanced Raman Scattering (SERS) active gold nanostars represent an opportunity in the field of bioimaging and drug delivery. The combination of gold surface chemical versatility with the possibility to tune the optical properties changing the nanoparticles shape constitutes a multimodal approach for the investigation of the behavior of these carriers inside living cells. In this work, SERS active star-shaped nanoparticles were functionalized with doxorubicin molecules and covered with immuno-mimetic thiolated polyethylene glycol (PEG). Doxorubicin-conjugate gold nanoparticles show an intense Raman enhancement, a good stability in physiological conditions, and a low cytotoxicity. The strong adsorption of the anticancer drug doxorubicin in close contact with the gold nanostars surface enables their use as SERS tag imaging probes in vivo. Upon laser irradiation of the nanoparticles, a strong SERS signal is generated by the doxorubicin molecules close to the nanostars surface, enabling the localization of the nanoparticles inside the cells. After long time irradiation, the SERS signal drops, indicating the thermally driven delivery of the drug inside the cell. Therefore, the combination of SERS and laser scanning confocal microscopy is a powerful technique for the real-time analysis of drug release in living cells.

## 1. Introduction

Nanoparticle formulations for applications like imaging or drug delivery is currently a very attractive area of research [1,2,3]. In the last years, a plethora of nanomaterials have been investigated for the synthesis of optically active contrast agents for imaging applications [4,5,6,7,8]. In this field of research, gold nanoparticles have emerged as one of the best candidates for the production of biocompatible, optically active, and functionalized nanocarriers and nanotags [9,10,11]. 

The aspect that elevates gold nanoparticles above the other metal stems from the possibility to finely control the size and shape of the nanoformulation. 

In addition, gold surface can be easily functionalized with protective and anti-fouling polymers, drugs, fluorophores, etc. [12,13]. Gold nanoparticles (AuNP) display a strong absorption in the range from visible to NIR frequencies resulting from their surface plasmon resonance. For this reason, AuNP are widely used as optical contrast agents with different imaging techniques including Surface Enhanced Raman Scattering [14], Two Photon Fluorescence Imaging [15], and Photoacoustic Tomography [16]. Besides imaging applications, photothermal therapy and photoassisted drug delivery based on AuNP have been also reported [17,18,19,20,21].

Among all the gold nanostructures, gold nanostars (AuNS) are one of the most interesting for nanophotonic applications. The “morning star”-like shape of these nanoparticles is composed of a central sphere surrounded by gold tips, concentrating the electromagnetic field at their extremity. This particular geometry induces a shift of their localized surface plasmon resonances (LSPRs) frequency in the near infrared (NIR) region [22]. This, combined withthe intensification of the electromagnetic field at the tips makes AuNS highly efficient optical antennas for the amplification of the Raman scattering of molecules placed in close contact with the gold surface, a process known like Surface Enhanced Raman Scattering. Metallic nanoparticles supporting LSPR coupled to specific organic Raman reporter molecules represent a novel class of nanoprobes named “SERS tags” [23]. The advantage of using SERS tags in biological application stems from their photostability, the simple mechanism of generating a multitude of unique spectral signatures, and the possibility to be excited at any light wavelength. It was reported that Raman tags based on gold nanoparticles have an emission intensity of about two orders of magnitude higher with respect to traditional NIR quantum dots. 

Irradiation of plasmonic nanoparticles could induce a sensible increase of the local temperature because of the conversion of radiative energy into heat. This was deeply exploited in the past for photothermal therapy applications. More recently this effect has been used to boost the release of substances from cargos placed closely to the surface of plasmonic nanoparticles [24]. In principle, photothermally controlled drug release could be used to realize chemotherapeutical treatments that are both spatially and temporally controlled. In this therapy, the drug is stably bonded to a nanocarrier avoiding unwanted drug release until the particles reach the target site. Then, the irradiation of the area induces the fast release of the drug in a controlled mode.

Herein, we design a new strategy to enhance the analysis of the mechanism behind the drug release both via the SERS imaging and the light triggered drug release.

AuNS (60 nm) capped with thiolated PEG and functionalized with doxorubicin was synthesized and used as optical labels inside living A549 cancer cells. Doxorubicin, being a positively charged molecule can be readily adsorbed on the surface of the AuNS. The close proximity of the molecule to the gold surface generates a strong SERS signal that can be used to track the nanoparticles fate inside the cells. In addition, prolonged irradiation of the nanoparticles causes a progressive decrease of the Doxorubicin Raman signal, indicating the photothermal-driven diffusion of the drug from the nanoparticles to the cellular environment. Thanks to the high amplification of the Raman signal, this peculiar system allows the real-time, high resolution, and ultrafast investigation of the drug release inside living cells. This could open new opportunities for the creation of real time bioimaging and biosensing optical nanoprobes for in vivo investigations. 

## 2. Materials and Methods

### 2.1. Synthesis of AuNS-Dox-PEG

The 60 nm Gold nanostars were supplied by Immagina Biotechnology. Briefly, 100 mM hydroxylamine solution in 0.01 M sodium hydroxide was rapidly added to 1 mL of a 1 mM HAuCl_4_ solution in water under stirring. The solution color turned quickly from transparent to deep green. The nanoparticles were purified by three centrifugation-washing cycles [25]. Gold nanostars were incubated in 1 mL of 0.1 mg/mL thiol-terminated-PEG solution in water overnight. Then the nanoparticles were purified by at least 6 centrifugation-washing cycles (AuNS-PEG).

PEG-capped gold nanostars were incubated with 10^−^^5^ M Doxorubicin solution under magnetic stirring for 5 h. The nanoparticles were purified by five centrifugation-washing cycles until the disappearance of the Doxorubicin luminescence in the supernatant.

### 2.2. Cell Culture

Commercial A549 human lung carcinoma epithelial cell line (ATCC, CCL-185™) was cultured in RMPI-1640 medium (Sigma R7509) supplemented with 10% FBS and 4 mM glutamine. The cells were incubated at 37 °C in a 5% CO_2_ atmosphere.

### 2.3. Cell Viability

A total of 10,000 A549 cells were placed into a 96-well plate and placed in incubation until the cells reached 80% of confluence. The culture medium was then discarded, and the cells were incubated with fresh medium as control or by fresh medium containing Doxorubicin or of the AuNS at different concentrations for 24 or 48 h. The viability was measured by Trypan Blue Exclusion protocol.

### 2.4. Cell Uptake and Drug Release Investigations of AuNS-Dox-PEG

A total of 50,000 A549 cells were incubated for 2 days in a 18-mm glass coverslips. The cells were incubated with 200 µL of 0.1 mg/mL AuNS-Dox-PEG nanoparticles for 1 and 2 h and then analyzed by laser scanning confocal microscopy.

### 2.5. Characterization

UV–VIS absorption measurements were obtained using a Cary 500UV–visible–near infrared spectrophotometer. TEM images were acquired using a Philips CM12 TEM operated at 120 kV. SEM images were acquired with a JSM-7001F instrument. Hydrodynamic diameters were measured utilizing a Malvern Zetasizer Nano ZS dynamic light scattering (DLS) in back scattering mode at 25 °C. Measurements were performed in triplicate on equivalent samples. Laser scanning confocal microscopy (LSCM) analysis was carried out using a Leica 200D microscopy. Doxorubicin emission was measured using an excitation wavelength of 488 nm and an acquisition range from 500 to 530 nm. Gold nanoparticles scattering was studied using a 633 nm excitation wavelength and an acquisition range from 620 to 650 nm. Raman spectra were acquired between 300 and 3000 cm^−^^1^ utilizing a Horiba–Jobin–Yvon microscopy coupled to of a 20 mW He–Ne laser operating at 633 nm. The laser spot size was about1 µm. The resolution was about 0.35 cm^−^^1^/pixel. 

### 2.6. Modelling 

The simulated extinction spectra of the Gold nanostars were calculated using the Discrete Dipole Approximation method (DAA). The simulated system was composed by a central sphere with a radius of 40 nm and two conical tips with a length of 10 nm and a tip curvature of 4 nm. Gold dielectric function was obtained using the experimental values of Johnson and Christy [26]. 

## 3. Results and Discussions

**Figure 1:**
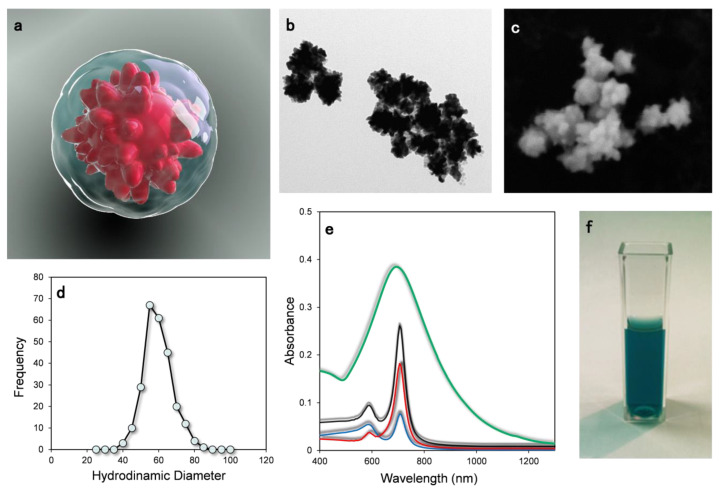
(**a**) 3D representation of a gold nanostar functionalized with doxorubicin molecules and coated thiol-PEG. (**b**) TEM and (**c**) SEM images of 60 nm gold nanostars. (**d**) Gold nanostars size distribution obtained by Dynamic Light scattering Scattering analysis. (**e**) UV–VIS absorption spectroscopy (green continuous line), simulation of total extinction spectrum (black line) of AuNS. The contribution of the calculated absorption (blue line) and scattering (red line) were also plotted. (**f**) Photograph of AuNS water suspension.

In Figure 1a, the 3D representation of a 60 nm gold nanostar functionalized with doxorubicin (Dox) and capped with PEG polymer is presented. Thanks to the semi-covalent interaction between gold and thiol groups at the end of the polymer chain, PEG can be readily adsorbed by the gold nanoparticles providing a protective shell around the nanoparticles. Then, Doxorubicin molecules can penetrate this shell and be adsorbed on the surface of the gold nanostars. The chemical interaction between the positively charged Dox functional group (a primary amine) and the gold NP ensures a stable bonding between the molecule and the gold surface.

In Figure 1b,c, representative transmission and scanning electron microscopy images of the AuNS are shown. The NPs showed a “morning star” geometry with conical tips on the surface and a mean size (tip to tip) of about 60 nm. The statistical size distribution calculated by Dynamic Light Scattering indicates an average size of 63 ± 8 nm (Figure 1d). UV–VIS absorption spectroscopy is reported in Figure 1e. The spectrum displays a broad absorption band centered at about 710 nm that falls into the NIR region. To simulate the optical response of these nanostructures, numerical modeling was carried out by using the Discrete Dipole Approximation. The extinction cross section was calculated as the sum of absorption and scattering contributions. The simulated total extinction spectrum (black line) is composed by an intense component at around 740 nm assigned to the dipolar resonance localized at the nanoparticles’ tips. The shoulder at about 600 nm is attributed to a mixture of quadrupole and octupole modes.

**Figure 2:**
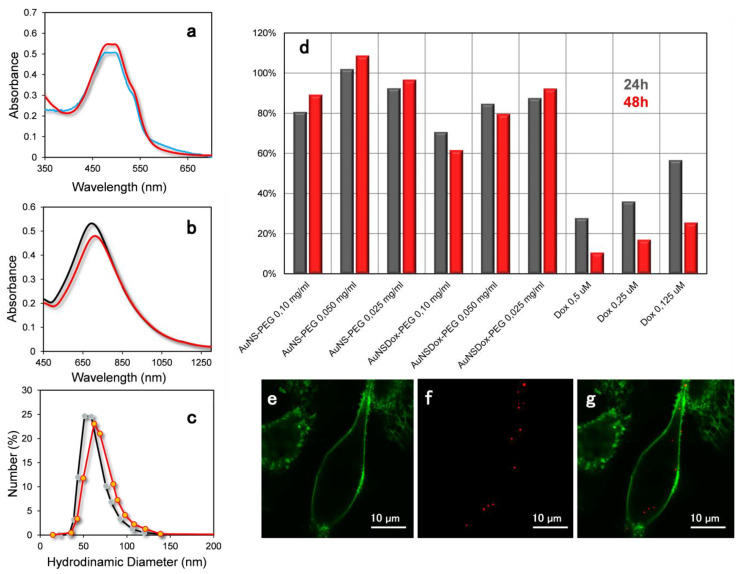
(**a**) UV–VIS spectra of Doxorubicin solution (red line) and supernatant obtained by the separation of the nanoparticles after 5 hrs of incubation with AuNS-PEG (blue line). (**b**) UV–VIS adsorption spectra of AuNS-Dox-PEG nanoparticles incubated in water (black line) and in PBS pH 7.4 (red line). (**c**) Dynamic Light Scattering analysis of AuNS-Dox-PEG nanoparticles incubated in water (black line) and in PBS pH 7.4 (red line). (**d**) Viability of A549 cell line incubated for 24 and 48 h with free doxorubicin, AuNS-PEG and AuNS-Dox-PEG at different concentrations (expressed in mass concentration of nanoparticles). Viability is expressed as fraction of the control (cells with complete medium). (**e**–**g**) Colocalization of gold nanoparticles inside living cell by LSCM. Green: fluorescence of the membrane stained with WGA-Alexa488. Red: Scattering of gold nanostars.

Gold nanostars were further capped with thiolated PEG and functionalized with doxorubicin. After incubation with the drug, the nanoparticles were precipitated by centrifugation and the supernatant was analyzed by UV–VIS absorption. The spectrum of the supernatant was compared with that of a doxorubicin water solution at the same concentration used for the loading on the AuNS. The spectra are reported in Figure 2a. The supernatant spectrum shows a slightly reduced doxorubicin absorption peak, and this confirms. This indicates the effective loading of Dox molecules on the AuNS. The doxorubicin uptake was estimated to be around 1.2% w_drug_/w_AuNS_ from the UV–VIS spectrum. The stability of the AuNS-Dox-PEG nanoparticles was investigated in PBS buffer, a good surrogate of the cellular medium. Nanoparticles UV–VIS absorption and hydrodynamic diameter in high salt buffer (red plots) did not show remarkable differences compared to the same values measured in Milli-Q water (black plots) (Figure 2b,c). This indicated that the PEG coating can efficiently shield the nanoparticles from the medium, inducing a high stability to the nanoformulation.

The cytotoxicity of the AuNS-PEG and AuNS-Dox-PEG nanoparticles was investigated in A549 cells by Trypan Blue assay (Figure 2d). The cells were incubated for 24 h and 48 h with AuNS-Dox-PEG, AuNS-PEG, or Dox. AuNS-PEG did not display any cytotoxic effect to cells even after 48 h of incubation at a concentration of 0.1 mg/mL. This result is in agreement with other papers reporting a very low cytotoxicity of PEG coated nanoparticles [27]. The AuNS-Dox-PEG nanoparticles showed a lower cytotoxicity respect to the free Dox, due to the partial release of the drug during the incubation. Figure 2b–d displays the laser scanning confocal analysis on A549 cell line incubated with AuNS-PEG nanoparticles for 1 h. The membrane was stained with WGA-Alexa488 (excitation 488 nm, emission 520 nm). Gold scattering was obtained by excitation at 633 nm and an acquisition band of 620–650 nm. This excitation wavelength was selected considering the optical properties of the gold colloids. AuNS display the classical broad band associated to surface plasmon resonance with an absorption maximum at around 680–720 nm. When the excitation wavelength is in the blue region (480 nm), the WGA-Alexa488 luminescence at 520 nm is predominant compared to the Raman gold scattering. On the contrary, at 633 nm the excitation energy falls out of the absorption band of the dye but can excite plasmon modes of the gold nanostars. The isolated nanoparticles dimensions (around 60 nm) are much smaller respect to the diffraction limits of the visible light (around 500 nm) that represents the higher resolution that can be obtained by the optical imaging. The spots presented in the figure have a dimension of around 1µm or greater and indicate the presence of nanoparticle aggregates inside the cell. This is in agreement with works reported in the literature showing that nanoparticles of this dimensions are normally present as aggregates in endosome compartments of the cell [28].

**Figure 3:**
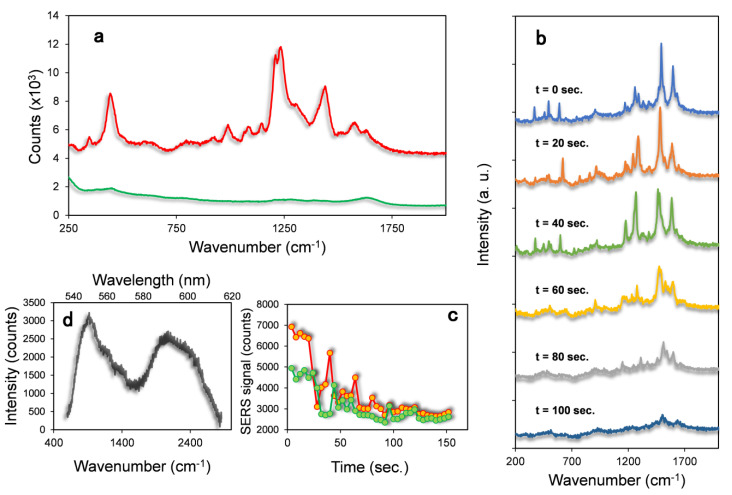
(**a**) Raman spectroscopy of the AuNS-Dox-PEG sample (red line) and Doxorubicin solution (green line) using 633 nm laser excitation in water. (**b**) Raman spectra of living cells incubated with AuNS-Dox-PEG acquired at different time intervals under continuous irradiation from top (t = 0 s) to bottom (t = 100 s). (**c**) Plot of the integrated area of the 1500 cm ^−^^1^ (red dots) and 1600 cm^−^^1^ (green dots) Raman peaks corresponding to the quinine ring and C=C bond vibrations of Dox molecules. (**d**) Doxorubicin Fluorescence emission of the hotspot acquired after the Raman measurements.

In Figure 3a, the Raman spectroscopy of the AuNS-Dox-PEG nanoparticles in water is reported. The spectrum of the sample shows some peaks in the region 300–2000 cm^−^^1^ that are attributed to the Raman vibration of the doxorubicin molecules adsorbed on the gold nanoparticles. For comparison, 10^−^^5^ M doxorubicin water solution does not show any peak in this range (Figure 3a, green line). The estimated maximum concentration of the doxorubicin molecules in the AuNS-Dox-PEG solution is 10^−^^8^ M, a value that is about three orders of magnitude lower than that of the doxorubicin solution. The higher intensity of the Raman peaks for the AuNS-Dox-PEG in respect to the free doxorubicin solution is due to the enhancement of the Raman scattering induced by AuNS. In Figure 3b, the Raman analysis of the A549 cell incubated with the AuNS-Dox-PEG nanoparticles for 1 h is reported. The Raman analysis of the hotspot shows intense emission typical of the doxorubicin phonon transitions. The spectra were acquired in the same region with a continuous irradiation and 4 s. of integration time. The modulation of the peaks intensity and position could be correlated to structural changes of the molecules near the nanoparticles. As it appears in Figure 3b, the intensity of the Raman peaks is not constant with time. By taking as references the peak at around 1500 cm^−^^1^ assigned to the stretching of quinine ring of doxorubicin and the peak at 1600 cm^−^^1^ assigned to the stretching of C=C bonds [29], we can follow the change in intensity of the doxorubicin Raman signal deriving from the Dox molecules. The plot of the integrated area of the peaks at 1500 and 1600 cm^−^^1^ as a function of time reported in Figure 3c shows a continue decrease of the signal intensity. This effect can be explained with a modification of the position of the Dox molecules on the gold surface caused by the local increase of the temperature. The latter is due to the continue laser irradiation promoting the drug release from the AuNS with a consequent decrease of the Raman intensity. This photothermal release is confirmed by the appearance of the doxorubicin fluorescence emission after the Raman analysis (Figure 3d). Doxorubicin fluorescence is quenched when the molecule is adsorbed on the gold colloid. This effect is caused by the interference of the AuNS plasmonic field with the radiative decay rate of fluorophores closely associated to the nanoparticles. This effect decreases when the distance between the emission center and the gold surface increases. As a matter of fact, during the diffusion of the doxorubicin from the gold nanostars, the Dox Raman intensity decreases while its fluorescence emission increases, in agreement with what is observed in our experiment. To further confirm the release of the drug from the cargos, cells incubated with AuNS-Dox-PEG were analyzed by laser Scanning Confocal Microscope possessing a high lateral resolution thus allowing spatially resolved fluorescence and Raman spectroscopy.

**Figure 4:**
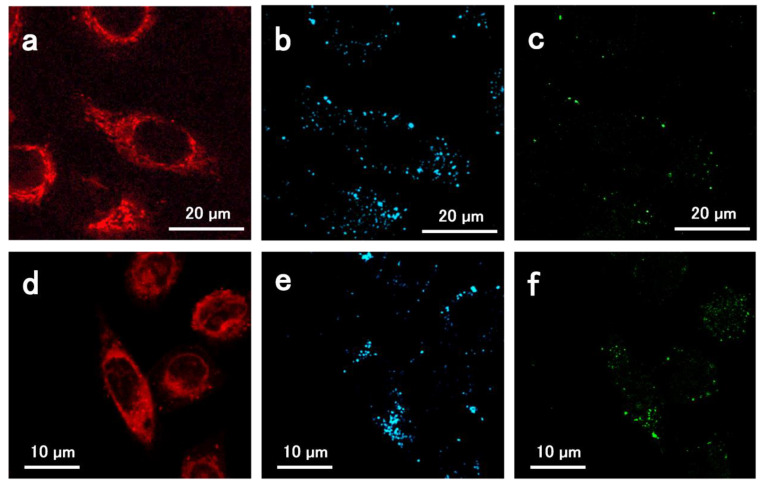
Confocal image of the AuNS-Dox-PEG sample incubated in the A549 cell for 1 h (**a**–**c**) and 2 h (**d**–**f**) using 488 nm (**a**,**d**) and 633 nm (**b**,**c**,**e**,**f**) laser excitation. (Red: doxorubicin fluorescence; blue: gold scattering; green: Raman emission).

In Figure 4, the LSCM images of A549 cell line incubated for 1h with AuNS-Dox-PEG NPs are reported. Red color is associated to doxorubicin luminescence (range 520–600 nm); blue spots are the AuNS scattering obtained by excitation at 633 nm and acquisition band of 620–650 nm. Finally, Raman emissions are labelled in green (excitation 633 nm, acquisition 640–720 nm). LSCM allows the localization of the Raman “hot spots” (green) inside the cell as well as unbound doxorubicin (in red) and the localization of the gold aggregates (blue). As expected, the green spots are partially superimposed on the blue ones. This because the highest enhancement of Raman scattering is expected from the gold nanoparticle aggregates, rather than from single isolated gold nanoparticles which cannot be detected by LSCM.

## 4. Conclusions

Gold nanostars (60 nm) functionalized with doxorubicin and capped with thiolated PEG were produced by an easy and reproducible chemical route. Raman spectrum shows the presence of intense and well resolved components deriving from the Doxorubicin molecules adsorbed on the gold nanostars surface. This system was adopted to investigate the nanoparticles localization inside tumor cells as well as to give information about the doxorubicin delivery in living cells. The drug release kinetic was studied analyzing both the fluorescence and the SERS signal intensities deriving from doxorubicin molecules without the need of additional fluorescent probes. Thanks to the small dimension of the final composite, AuNS are easily internalized in A549 living cells. The possibility to precisely localize the AuNS coupled to the intense Raman signal induced by their plasmonic resonance and their biocompatibility make these nanosystems a very promising tool for in vitro imaging applications.
